# Relationships among Leisure Physical Activity, Sedentary Lifestyle, Physical Fitness, and Happiness in Adults 65 Years or Older in Taiwan

**DOI:** 10.3390/ijerph17145235

**Published:** 2020-07-20

**Authors:** Yi-Tien Lin, Mingchih Chen, Chien-Chang Ho, Tian-Shyug Lee

**Affiliations:** 1 Graduate Institute of Business Administration, Fu Jen Catholic University, New Taipei City 242, Taiwan; sweet.oc18@gmail.com (Y.-T.L.); 081438@mail.fju.edu.tw (M.C.); 2 Artificial Intelligence Development Center, Fu Jen Catholic University, New Taipei City 242, Taiwan; 3 Department of Physical Education, Fu Jen Catholic University, New Taipei City 242, Taiwan; 093703@mail.fju.edu.tw; 4 Research and Development Center for Physical Education, Health, and Information Technology, Fu Jen Catholic University, New Taipei City 242, Taiwan

**Keywords:** physical activity, exercise, sedentary lifestyle, functional fitness, well-being, elderly

## Abstract

The purpose of this study is to understand the relationship among leisure physical activity, sedentary lifestyle, physical fitness, and happiness in healthy elderly adults aged over 65 years old in Taiwan. Data were recruited from the National Physical Fitness Survey in Taiwan, which was proposed in the Project on the Establishment of Physical Fitness Testing Stations by the Sports Administration of the Ministry of Education. Participants were recruited from fitness testing stations set up in 22 counties and cities from October 2015 to May 2016. A total of 20,111 healthy older adults aged 65–102 years were recruited as research participants. The fitness testing procedure was described to all participants, who were provided with a standardized structured questionnaire. Participants’ data included sex, city or county of residence, living status (living together with others or living alone), education level, and income. Physical fitness testing was conducted in accordance with The Fitness Guide for Older Adults published by the Sports Administration of the Ministry of Education. The testing involved cardiorespiratory endurance, muscle strength, muscle endurance, flexibility, balance, and body composition. The *t*-test was used to evaluate the differences between continuous and grade variables under the two classification variables of sex, city or county of residence, and living status. We used the MARS (multivariate adaptive regression splines) model to analyze the effects of physical fitness variables and leisure physical activity variables on happiness. Among healthy elderly adults, sex, age, living status, body mass index, and leisure physical activity habits proved to be related to happiness. Aerobic endurance (2-min step test), muscular strength and endurance (30-s arm curl and 30-s chair stand tests), flexibility (back stretch and chair sit-and-reach tests), and balance ability (8-foot up-and-go tests and one-leg stance with eyes open tests) were found to be related to happiness. The results of this study indicate that increased physical activity and intensity, as well as physical fitness performance, are associated with improved happiness.

## 1. Introduction

Population aging has becoming a focus of public health concerns worldwide, and the number of elderly adults is increasing in Taiwan. Although aging is an inevitable process in human life, it is considered an issue if it causes unfavorable functionalities. Population aging is currently challenging the public health care system and medical support of the communities. According to the World Health Organization (WHO), a region or country is considered an aged society if its older adults (aged 65 years and above) account for more than 7% of the total population; a super-aged society is indicated when this proportion reaches 20% [1]. Taiwan has been an aging society since 1993, and became an aged society in December 2018, with 14.56% of the population across the country classified as elderly. According to the Taiwan National Development Council [2], the proportion of elderly adults in Taiwan is projected to increase to 41.0% by 2061. This suggests a heavy burden on the productive population to provide for their dependents [3,4,5,6]. Thus, success aging is an essential topic for modern societies, particularly in Taiwan.

Basically, there are three elements to achieve success aging [7]. The first is reducing the risk of chronic diseases and disabilities. The second is to maintain cognitive and physical functions. The last is actively participating in society. In other words, maintaining quality of late life is the main purpose of success aging [8], and it includes multidimensional aspects, such as overall health status [9], biophysical functionalities [10], social relationships [11], and happiness [12]. Nevertheless, these dimensions are usually correlated with an elderly person’s physical fitness and daily activities. For example, physically fit elderly adults have reported that significant correlations with physical and social functioning, positive emotional enjoyment, mental satisfaction, and overall life experience create an improved quality of life compared with those who are unfit [13,14,15,16]. Physical activity and fitness level play a fundamental role for elderly adults in avoiding diseases and disabilities, maintaining daily functions, and participating in society. Although human functions are deteriorating gradually, there are still approaches that inhibit the process of degeneration, maintain social functioning and quality of life, and enable independent living [17].

On the other hand, a sedentary lifestyle is becoming a serious problem for modern societies and increasing the process of human aging [18]. Insufficient physical activity has been associated with unfavorable cardiorespiratory functions, as well as increased risks of chronic diseases [19,20,21]. Furthermore, a sedentary lifestyle may also cause muscle deterioration, reduce metabolism, lead to obesity, impair cognitive function, and damage mental health [22]. Elderly adults are commonly sedentary. Therefore, maintaining physical activity is crucial, especially for elderly adults. Leisure time physical activities (LTPAs) have been indicated as a common approach to delaying the negative impacts from aging [23]. It is well documented that elderly adults participating in LTPA regularly tend to be heathier than those who are sedentary [15,24,25]. Physically fit elderly adults are usually able to self-care in their daily life [26]. Moreover, participating in LTPA in a social environment has been positively associated with one’s social well-being. Previous studies have reported a significant association between LTPA and happiness among elderly individuals, through its influence on emotions, mental health, and self-efficacy [27,28,29]. Further, LTPA has been indicated as alleviating depression and anxiety [30,31,32]. Accordingly, studies have revealed that LTPA is associated with positive life satisfaction and overall happiness, which is critically affecting the quality of elderly adults’ late life.

Happiness is an overall evaluation of life experience and wellbeing [33], which is important for elderly adults. However, although current studies have provided evidence to support the association between LTPA participation and happiness [34], the relationships between physical fitness performance, happiness, and lifestyle still require further study, especially in Taiwan. A larger study sample among Taiwanese elderly adults is needed for an improved understanding of the relationship between physical fitness performance and happiness.

Therefore, this study aimed to understand these relationships among healthy elderly adults in Taiwan. A representative database and the multivariate adaptive regression splines (MARS) were applied in this study.

## 2. Materials and Methods

This study adopted a cross-sectional design and employed the National Physical Fitness Survey in Taiwan, which was proposed in the Project on the Establishment of Physical Fitness Testing Stations by the Sports Administration of the Ministry of Education. Participants were recruited from fitness testing stations set up in 22 counties and cities from October 2015 to May 2016. All participants were fully informed about the study and provided their informed consent. Additionally, the study design and analysis were approved by the Institutional Review Board of Fu Jen Catholic University Hospital (FJU-IRB C108006). Initially, 20,111 healthy older adults aged 65–102 years were recruited as research participants.

The inclusion criteria for older adults were as follows: (1) 65 years or older and (2) no heart disease, hypertension, chest pain, dizziness, bone-related conditions, or joint-related conditions. The exclusion criteria were as follows: (1) having a resting heart rate >100 beats/min; (2) having a resting systolic blood pressure >140 mm Hg and a resting diastolic blood pressure >90 mmHg; and (3) receiving antihypertensive medication. Finally, 13,703 participants (4978 men and 8725 women) were recruited, and secondary data were obtained for preliminary analysis. Subsequently, the fitness testing procedure was described to all participants, who provided informed consent before the testing began.

First, an analysis was conducted on the secondary fitness testing data from the Physical Fitness Database of the Sports Administration of the Ministry of Education. Before the fitness testing began, participants were provided with a standardized structured questionnaire. The variables, sections, and ratings of the questionnaire are explained as follows:

1. Demographic information: age, sex, living status (living together with others or living alone), education level, and income.
2. Physical fitness testing: Testing was conducted in accordance with The Fitness Guide for Older Adults published by the Sports Administration of the Ministry of Education. The testing involved cardiorespiratory endurance, muscle strength, muscle endurance, flexibility, balance, and body composition. The method and procedure for each test item are described as follows [35]:
  I. Aerobic endurance (2-min step tests): Colored tape was used to mark the height of the middle point between the patella and iliac spine on a wall. This indicated the height to which the participant was asked to raise their knees as they walked in place. The number of steps within 2 min was recorded.
  II. Muscle strength and endurance in the upper extremities (30-s arm curl): Each participant was asked to sit on the edge of a chair on the side of their dominant hand with their back straight and both feet fully touching the floor. They were then asked to hold a dumbbell with their dominant hand and bend their elbow toward their shoulder. The number of curls (bending the elbow toward the shoulder and returning to the original position) within 30 s was recorded.
  III. Muscle strength and endurance in the lower extremities (30-s chair stand tests): Each participant was asked to sit in the center of a chair with both feet touching the floor and arms crossed in front of their chest. The number of times each participant stood up from a seated position within 30 s was recorded.
  IV. Flexibility in the upper extremities (back stretch tests): Each participant was asked to place their dominant hand behind their shoulder on the same side with their palm facing their back; they were then asked to reach their other hand (with the palm facing away from their back) from the lower back upward toward their dominant hand until the two hands overlapped or until they could not reach any further. The test result was determined based on the distance between the two middle fingers. Participants received negative points for the distance between the fingers if the hands did not touch and received positive points for any overlapping.
  V. Flexibility in the lower extremities (chair sit-and-reach tests): Each participant was asked to bend one leg, straighten the other leg with the heel touching the floor, place their hands on top of each other, reach the hands toward the toes of their strengthened leg, and hold the position for 2 s. This was repeated twice per leg. Participants received negative points for the distance between their fingertips and the tips of their toes, and they received positive points for the distance of their fingertips beyond their toes. The highest score for each participant was recorded as the test result.
  VI. Dynamic balance ability (8-foot up-and-go tests): Each participant was seated on a chair with a 2.44 m traffic cone in front of them. When the tester said “start,” they were asked to walk as fast as possible around the traffic cone (running was not allowed), walk back, and sit back on the chair.
  VII. Static balance ability (one-leg stance with eyes open tests): Each participant was asked to stand with their hands on their waist, raise one leg, and then place it on the inner side of the ankle of the other leg. Each participant was alternately tested with both legs; full marks were given for a 120 s stance.
  VIII. Body composition: body mass index (BMI) and waist-to-hip ratio (WHR):
    (a) BMI is used to measure obesity and is calculated by dividing body weight (kg) by the square of height (m).
    (b) To measure WHR, each participant was asked to take a regular breath, after which their waist circumference was measured at the level of the middle point between the lower edge of the ribs and the ilium; hip circumference was measured around the widest part of the hips under the pelvic bone. Both the waist and hip circumferences were measured twice. WHR was calculated by dividing the waist circumference by the hip circumference; the mean of the two WHR measurements was recorded for each participant.
3. Physical activity investigation: The participants were asked whether they had participated in physical activities for at least 10 min during the preceding 7 days, primarily to determine whether they have a sedentary lifestyle. Indicators of a lifestyle considered sedentary or not include walking status, how laborious one’s physical activity is, and the extent to which one sits on a daily basis.
4. Happiness index: The happiness of elderly adults was assessed using data collected from the Project on the Establishment of Physical Fitness Testing Stations. In this study, a structured questionnaire (with face-to-face administration) was designed based on the Cantril ladder scale (from 0 to 10) in the 2012 Gallup World Poll. The items included “On a scale from 0 to 10, how happy were you yesterday?”

### Statistical Analysis

We used R software (version 3.5.3; R core team, Vienna, Austria) to analyze the data. The questionnaire included five questions related to self-rated happiness status, namely: happiness, self-rated health, worry, frustration, and life satisfaction. We used the scoring method for the general health questionnaire to integrate the scores of these five variables [36], which yielded a happiness score range of 0–50 points. The higher the score, the higher the happiness.

The independent samples *t*-test was used to compare the distributions of continuous and categorical variables, such as demographics, exercise habits, and physical fitness performance, based on binary classifications such as sex, living status, and living area. The *t*-test results are presented as mean ± standard deviation. We used the MARS model to analyze the effects of the physical fitness and lifestyle variables on happiness. Friedman and Roosen [37] first proposed the MARS model as a flexible procedure for modeling the continuity and discontinuity relationships that are nearly additive or involve interactions with few variables [38]. The model procedure was inspired by the recursive partitioning technique used in CART (classification and regression tree) and generalized additive modeling [39], thus resulting in a continuous model with continuous derivatives. This approach effectively identifies optimal variable transformations and interactions, which are complex data structures that can be difficult to locate in high-dimensional data; thus, the MARS model can effectively uncover important data patterns and hidden relationships [40]. In this study, the significance level of all the aforementioned statistical tests was set at *p* < 0.05.

## 3. Results

Table 1 shows the results of the *t*-test for the groups stratified by sex. The results revealed a significant difference in physical fitness performance, weekly labor, and physical activity habits. Among these, the chair sit-and-reach tests, back stretch test, and labor activity of the male and female groups exhibited the largest differences. Women had significantly higher flexibility than men (chair sit-and-reach tests: 5.86 vs. 0.92; back stretch tests: −5.26 vs. 12.12). Men had more weekly labor activity than women (142.6 vs. 95.9 h). The differences between groups stratified by living status and urbanization were small. However, weekly labor activity among participants who lived with others was clearly higher than that among solitary participants. The analysis and verification results are shown in the Appendix A (Table A1 and Table A2). Based on these results, we chose to apply the MARS model separately for both sexes.

The regression coefficient of the model was estimated using the MARS method. The MARS method was used to select significant variables from the high-dimensional set of variables, after which a piecewise estimation process was implemented. The results are shown in Table 2, which was sorted according to the degree of influence of the variables.

**Figure 1:**
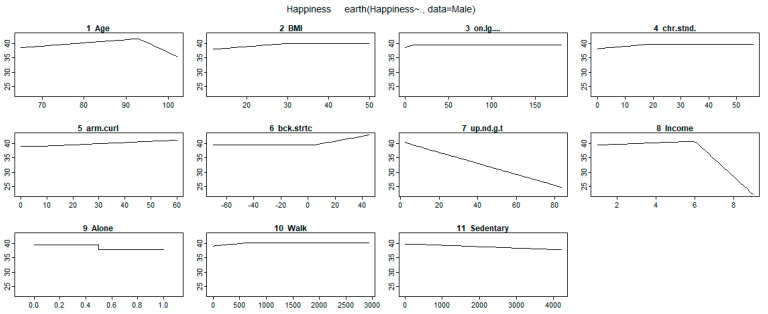
Trends of the effects of physical fitness and lifestyle variables on happiness among male participants. BMI—body mass index.

**Figure 2:**
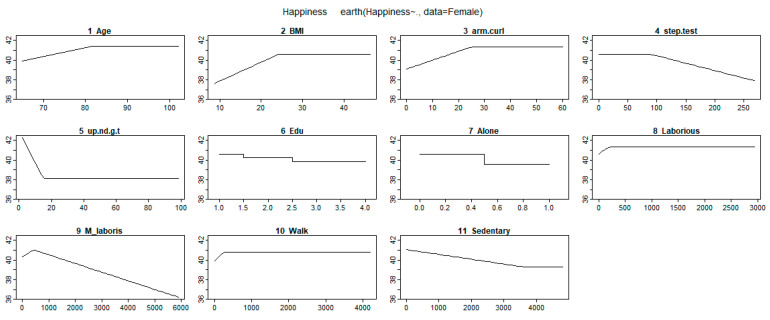
Trends of the effects of physical fitness and lifestyle variables on happiness among female participants. BMI—body mass index.

Other variables removed by the MARS model included height, weight, waist circumference, hip circumference, WHR, and the sit-and-reach test. These variables had no effect on happiness. The response trend figures for the selected variables and coefficients in Table 2 are shown in Figure 1 and Figure 2 for male and female participants, respectively.

The trend analysis of the piecewise estimation is shown in Figure 1 and Figure 2. Based on Figure 1 and Figure 2 and Table 2 for male and female participants, according to the comparison between intercepts and coefficients estimated by MARS model, we found that living status had the most significant impact on the estimation of happiness. The coefficients of solitary living in the male and female groups were −1.70 and −1.04, respectively. The second important impact factor was age, and the age of 93 years was the optimal age of happiness among male participants. Based on the slope of the coefficient, happiness increased slowly from the age of 65 to 93 years, after which happiness rapidly declined. The age knot among female participants was 82 years, and happiness was not affected by ages older than 82 years. The third important impact factor was BMI. For the male and female groups, participants with low BMI had low happiness. When the BMI was greater than the knot (male: 28.9, female: 24.1), it did not affect happiness. Education level only affected happiness among women; those with a level of education below university had lower happiness. Income status only affected happiness among men; for monthly incomes of more than $20,000 NTD (New Taiwan Dollar), the happiness level slightly increased to $60,000 NTD and then rapidly decreased.

Among the physical fitness variables, one-leg stance with eyes open test results had limited effects on happiness. This variable negatively affected happiness among men only when their performance value was less than 9. The chair stand test results also had a weak negative effect on happiness among men when its value was less than 18. When the backstretch test result was higher than 4 among men, the happiness increased. When the bicep curl test results were less than the knot of 25, the effect on happiness was negative among women; however, it had no effect on happiness when its value was higher than the knot. For the standing knee lift performance (representing cardiopulmonary function) among women, happiness was negatively affected for values higher than 88. As the (8-foot up-and-go tests) increased, the happiness of male participants decreased. The effect of 8-foot up-and-go tests in the female group was evidently negative when performance was less than the knot of 15; however, 8-foot up-and-go tests had no effect on happiness when its value was higher than the knot.

Among the lifestyle variables, labor activity, and moderate labor activity only affected happiness in the female group. When the period of moderate labor activity was more than 210 h, it had a negative effect on happiness. Weekly walking and sedentary hours also affected the happiness among both men and women.

## 4. Discussion

The purpose of the present study was mainly to investigate the relationship between physical fitness and happiness status among Taiwanese elderly adults. Demographic variables, body composition variables, physical fitness variables, and daily physical activity habits were analyzed as independent variables, and happiness indicators were adopted as dependent variables using the MARS method. Differences were found between the ages and sexes. Each category of variables influenced happiness among older adults. Public health research using the MARS model is rare, but more detailed segmentation regression results can be obtained.

This survey was based on an official test conducted by the Ministry of Education in Taiwan. All physical fitness tests were administered by trained examiners. A standardized training instruction manual, which included detailed procedures for measuring various fitness indicators, was provided to the trained examiners. In addition, after the physical fitness tests, a standardized questionnaire was administered by trained interviewers. The trained interviewers supervised and assisted participants in completing the questionnaire. Therefore, we could guarantee that all observations were recorded in the same way, thus ensuring interobserver and intra-observer reliability.

In our study, BMI was considered as a risk factor for obesity. BMI is not a direct measurement of body fat, and BMI ranges did not represent the level of obesity. BMI is an accessible measurement method, and it is suitable for characterizing the level of obesity in Taiwan. BMI cutoffs were suggested by Health Promotion Administration, Taiwan. In addition, studies have used BMI to characterize the level of obesity in Taiwan [41], indicating that BMI can be used to measure obesity among the residents of Taiwan. Most studies have shown that, as BMI increases, the risk of obesity increases. The aforementioned explanation illustrates the applicability of BMI to the assessment of obesity risk in Taiwan; the prevalence of obesity in Taiwan is closely correlated with BMI.

We applied the MARS model for each sex separately. Based on the discussion of body function, sex may have different effects on behavioral habits and physical fitness. Previous studies have demonstrated that body structure development, muscle strength growth, body fat change efficiency, energy intake patterns, and other aspects differ significantly between sexes [42,43,44,45]. Future studies should focus on the mechanisms among above-mentioned physical fitness performances and happiness.

Some limitations in the present study should be addressed. First, because a secondary database was used, other potential confounders, such as chronic diseases or mental disorders could not be discussed. Future studies should comprehensively investigate other potential confounders. Second, the cutoff age of elderly adults was 65 years and above; however, the WHO has also suggested “early old age” with a classification from 60 years old. Using population without age stratification, the analysis might have lacked some critical information. Future studies may investigate the differences among different periods of late adulthood. Third, the present study was conducted with a cross-sectional design. There is not a cause-and-effect relationship can be guaranteed.

## 5. Conclusions

This study revealed that age and living status are the most important factors affecting happiness. Sedentary lifestyle was found to have a small effect on happiness. Among the physical fitness variables, height, weight, and WHR (representing body composition) were found to have no significant effect on happiness; moreover, BMI and the six fitness indexes (i.e., the one-leg stance with eyes open tests, the chair sit-and-reach tests, the bicep curl test, the standing knee lift test, the back-scratch test, and the 8-foot up-and-go tests) had significant effects on happiness.

## Figures and Tables

**Table 1:** Results of *t*-tests for sex.

Variables	Male	Female	*p*-Value
Population	4978 (36%)	8725 (64%)	
Age	74.233	73.228	<0.001
Education	2.0474	1.5193	<0.001
Income	1.2949	1.1225	<0.001
BMI	24.835	24.913	0.20
WHR	0.92414	0.88042	<0.001
Stance with eyes open	13.621	11.241	<0.001
Chair stand	15.220	14.396	<0.001
Arm curl	17.945	17.225	<0.001
2-min step	85.288	81.910	<0.001
Chair sit-and-reach	0.92135	5.85516	<0.001
Back stretch	−12.1460	−5.2627	<0.001
Up-and-go	7.4862	7.8250	<0.001
Laborious	142.554	95.856	<0.001
M_laborious	258.07	229.73	<0.001
Walk	347.48	321.62	<0.001
Sedentary	1100.8	1090.3	0.40
Happiness	39.175	39.593	<0.001

Note. BMI—body mass index; WHR—waist-to-hip ratio.

**Table 2:** Analysis results for happiness based on the MARS model.

Variables	Male		Female	
	Selected Variable	Coefficient Estimation	Selected Variable	Coefficient Estimation
Intercept		43.08	-	38.92
Age	Max (0, age-93)	−0.69	-	-
	Max (0, 93-age)	−0.11	Max (0, 82-age)	−0.09
Living Alone	-	−1.70	-	−1.04
Education	-	-	-	-
	-	-	Max (0, 3-Edu)	0.39
Income	Max (0, Income-6)	−6.25	-	-
	Max (0, 6-Income)	−0.27	-	-
BMI	-		-	-
	Max (0, 28.9-BMI)	−0.11	Max (0, 24.3-BMI)	−0.19
Stance with eyes open	-		-	-
	Max (0, 9-Single_Legged)	−0.10	-	-
Chair stand	-	-	-	-
	Max (0, 18-Chair_Sitting)	−0.08	-	-
Arm curl	Max (0, Arm_Flexion-7)	0.04	-	-
	-	-	Max (0, 25-Arm_Flexion)	−0.90
2-min step	-	-	Max (0, Knees_Up-88)	−0.02
	-	-	-	-
Back stretch	Max (0, Scratch-4)	0.09	-	-
	-	-	-	-
Up-and-go	Max (0, Around-13.8)	−0.19	-	-
	Max (0, 13.8-Around)	0.20	Max (0, 15-Around)	0.32
Laborious	-	-	-	-
	-	-	Max (0, 210-Laborious)	0.003
M_laborious	-	-	Max (0, M_laborious-420)	−0.001
	-	-	Max (0, 420-M_laborious)	−0.002
Walk	-	-	-	
	Max (0, 630-Walk)	−0.002	Max (0, 270-Walk)	0.003
Sedentary	Max (0, Sedentary-450)	−0.001	-	-
	-	-	Max (0, 3600-Sedentary)	0.0005

Note. BMI—body mass index.

## Appendix A

**Table A1:** *t*-tests analysis based on living alone.

Variables	Not Living Alone	Living Alone	*p*-Value
Population	11280 (82%)	2423 (18%)	
Age	73.358	74.689	<0.001
Education	1.7389	1.5819	<0.001
Income	1.1932	1.1478	0.002
BMI	24.886	24.879	0.930
WHR	0.89634	0.89615	0.910
Step test	12.391	10.775	<0.001
Chair stand tests	14.813	14.147	<0.001
Arm curl	17.640	16.775	<0.001
One-leg stance	83.54	81.26	0.0004
Chair sit-and-reach	4.1156	3.8172	0.240
Back stretch	−7.6539	−8.2723	0.061
Up-and-go tests	7.6214	8.0769	<0.001
Laborious	115.855	98.695	0.004
M_laborious	243.79	222.49	0.001
Walk	330.47	333.57	0.72
Sedentary	1089.5	1115.8	0.10
Happiness	39.674	38.360	<0.001

Note. BMI—body mass index; WHR—waist-to-hip ratio.

**Table A2:** *t*-tests analysis based on living alone urbanization.

Variables	Rural	Urban	*p*-Value
Population	5699 (42%)	8004 (58%)	
Age	74.275	73.107	<0.001
Education	1.5113	1.8534	<0.001
Income	1.1481	1.2115	<0.001
BMI	25.054	24.765	<0.001
WHR	0.90328	0.89134	<0.001
Step test	10.570	13.199	<0.001
Chair stand tests	13.766	15.357	<0.001
Arm curl	16.693	18.052	<0.001
One-leg stance	78.211	86.645	<0.001
Chair sit-and-each	3.1120	4.7398	<0.001
Back stretch	−8.6306	−7.1457	<0.001
Up-and-go tests	8.2306	7.3255	<0.001
Laborious	114.29	111.77	0.610
M_laborious	233.34	244.78	0.084
Walk	315.94	341.75	<0.001
Sedentary	1067.5	1113.1	0.0002
Happiness	39.332	39.520	0.077

Note. BMI—body mass index; WHR—waist-to-hip ratio.
